# The Relationship between Generalized and Abdominal Obesity with Diabetic Kidney Disease in Type 2 Diabetes: A Multiethnic Asian Study and Meta-Analysis

**DOI:** 10.3390/nu10111685

**Published:** 2018-11-05

**Authors:** Ryan Eyn Kidd Man, Alfred Tau Liang Gan, Eva Katie Fenwick, Preeti Gupta, Mark Yu Zheng Wong, Tien Yin Wong, Gavin Siew Wei Tan, Boon Wee Teo, Charumathi Sabanayagam, Ecosse Luc Lamoureux

**Affiliations:** 1 Singapore Eye Research Institute, Singapore National Eye Centre, Singapore 169856, Singapore; man.eyn.kidd.ryan@seri.com.sg (R.E.K.M.); alfred.gan.t.l@seri.com.sg (A.T.L.G.); eva.fenwick@seri.com.sg (E.K.F.); preeti.gupta@seri.com.sg (P.G.); mwyzknight@yahoo.com.sg (M.Y.Z.W.); wong.tien.yin@singhealth.com.sg (T.Y.W.); charumathi.sabanayagam@seri.com.sg (C.S.); 2 Duke-NUS Medical School, Singapore 169857, Singapore; 3 Singapore National Eye Centre, Singapore 168751, Singapore; gavin.tan@singhealth.com.sg; 4 Department of Nephrology, University Medicine Cluster, National University Health System, Singapore 119074, Singapore; mdctbw@nus.edu.sg

**Keywords:** obesity, body mass index, waist-hip ratio, waist circumference, waist-height ratio, meta-analysis, diabetic kidney disease

## Abstract

This study examined the associations of body mass index (BMI), waist circumference (WC), waist-hip ratio (WHR) and waist-height ratio (WHtR) with diabetic kidney disease (DKD) in a clinical sample of Asian patients with type 2 diabetes (T2DM); substantiated with a meta-analysis of the above associations. We recruited 405 patients with T2DM (mean (standard deviation (SD)) age: 58 (7.5) years; 277 (68.4%) male; 203 (50.1%) with DKD) from a tertiary care centre in Singapore. DKD was defined as urinary albumin-creatinine ratio >3.3 mg/mmoL and/or estimated glomerular filtration rate <60 mL/min/1.73 m^2^. All exposures were analysed continuously and categorically (World Health Organization cut-points for BMI and WC; median for WHR and WHtR) with DKD using stepwise logistic regression models adjusted for traditional risk factors. Additionally, we synthesized the pooled odds ratio of 18 studies (*N* = 19,755) in a meta-analysis of the above relationships in T2DM. We found that overweight and obese persons (categorized using BMI) were more likely to have DKD compared to under/normal weight individuals, while no associations were found for abdominal obesity exposures. In meta-analyses however, all obesity parameters were associated with increased odds of DKD. The discordance in our abdominal obesity findings compared to the pooled analyses warrants further validation via longitudinal cohorts.

## 1. Introduction

Diabetic kidney disease (DKD), a serious microvascular complication of diabetes, is defined as decreased renal function (glomerular filtration rate (GFR)) with persistent clinically detectable proteinuria (albuminuria) [1]; and occurs in approximately 25–40% of patients with diabetes [2]. Given the dual problems of a significant risk of progression from DKD to end-stage renal disease (ESRD) [3], increased concomitant cardiovascular disease [4], and mortality [5], it is important to identify patients at risk of DKD, understand the underlying pathogenic pathways, and initiate renal and cardiovascular therapies based on the knowledge of these causal mechanisms.

Obesity is rapidly reaching epidemic proportions globally and Asia is no exception, particularly with the adoption of an increasingly westernized diet and sedentary lifestyle [6]. Obesity is an established risk factor for diabetes and hypertension [7,8], both linked with the development of DKD [9]. According to the World Health Organization (WHO) [10], there are two separate classifications of obesity namely ‘generalized’, defined as body mass index (BMI, calculated as weight in kilograms (kg) divided by height in meters (m) squared) of ≥30 kg/m^2^; and ‘central/abdominal’, assessed using waist circumference (WC) and/or waist-to-hip/height ratio (WHR/WHtR). While there is emerging evidence suggesting that both forms [11,12,13,14,15,16,17] contribute to the risk of DKD, independent of diabetes and/or hypertension, it is still unclear which one contributes more to the risk of DKD due to their close inter-relationship [18,19,20].

Our group has previously shown a differential association of BMI and WHR with diabetic retinopathy (DR), a visual microvascular complication of diabetes [21]. This lack of clarity is particularly problematic in patients with type 2 diabetes (T2DM) as up to half with impaired GFR have no overt albuminuria (non-albuminuric DKD) [22,23], which adds an additional level of uncertainty to the role of these two measures of obesity in the pathogenesis of DKD. In fact, recent research has advocated that GFR may be a better indicator of DKD, as urinary albumin-creatinine ratio (UACR) levels do not necessarily reflect actual levels of renal function [1].

In this study, we therefore examined the associations between generalized obesity (defined as BMI) and abdominal obesity (assessed using three different measures: WC, WHR and WHtR) with DKD (assessed using UACR and estimated GFR (eGFR)) in a well-characterized sample of Asian patients with T2DM. We also conducted a meta-analysis of studies evaluating the relationship of these two obesity indices with DKD in patients with T2DM to situate our findings in the broader context.

## 2. Materials and Methods

### 2.1. Study Population

Participants were recruited as part of the Singapore Diabetes Management Project (S-DMP), a clinic-based, cross-sectional study investigating the clinical, behavioural, and environmental barriers associated with optimal diabetes care in patients with diabetes [21]. In brief, we recruited 498 individuals aged ≥21 years, with Types 1 and 2 diabetes, from the Singapore National Eye Centre between 2010 to 2013. All participants had sufficient hearing enabling normal conversations, were not cognitively impaired (assessed using the 6 item Cognitive Impairment Test) [24], and lived independently in the community. Diabetes was physician-diagnosed, with the information retrieved from participants’ case notes. Written informed consent was obtained from all participants and the study was approved by the Singapore Centralized Institutional Review Board (Reference: 2010/470/A) and adhered to the tenets of the Declaration of Helsinki. For the current study, participants of Asian ethnicity (Chinese, Malays and Indians) with T2DM who had available eGFR data, as well as gradable retinal photographs and optical coherence tomography (OCT; Cirrus Version 3.0; Carl Zeiss Meditec, Jena, Germany) images of ≥6 signal strength to enable grading of diabetic retinopathy (DR) presence, a covariate in our multivariable adjusted model; (*N* = 405; comprising 303 Chinese, 35 Malays and 67 Indians) were included.

### 2.2. Assessment of Obesity Exposures

Participants were required to remove shoes and heavy objects such as belts, phones, keys, and wallets. Height was measured in centimeters (cm) using a wall-mounted measuring tape and weight in kg using a digital scale. BMI was calculated as weight in kg divided by the square of height in meters (kg/m^2^), and categorized into underweight (<18.5 kg/m^2^), normal (18.5–24.9 kg/m^2^), overweight (25–29.9 kg/m^2^), and obese (≥30 kg/m^2^) according to WHO-defined international BMI cut points [10], in order to maintain parity with previous studies in meta-analysis. However, due to the small sample size of individuals who were underweight (*N* = 3) and obese (*N* = 77), the underweight and normal weight categories, as well as those who were overweight and obese, were combined for analytical purposes.

Both waist and hip circumference values were assessed using a non-stretchable medical tape. Hip measurements (cm) were made at the maximal protuberance of the buttocks, while waist circumference (cm) was taken at the smallest horizontal girth between the costal margins and the iliac crests at the end of tidal expiration. Abdominal obesity was defined as WC >94 cm for males and 80 cm for females [25]; WHR as WC divided by the hip circumferences [21]; and WHtR as WC divided by height (in cm) [26]. Established WHR [25] and WHtR [27] categorizations were not utilized in the main analyses due to a need for a common unit of classification with previous WHR/WHtR and DKD studies when synthesizing data for the meta-analysis. We reran the analyses with established thresholds for the two exposures and the results are shown in Table S1.

### 2.3. Assessment of DKD

Serum creatinine was assessed using the Roche Integra 800 colorimetric assay (Roche Diagnostics Ltd., Rotkreuz, ZG, Switzerland), calibrated to the standards set by the National Institute of Standards and Technology (NIST). Estimated GFR (eGFR; in mL/min/1.73 m^2^) was calculated from serum creatinine using the Chronic Kidney Disease Epidemiology Collaboration (CKD-EPI) equation [28]. A mid-stream urine sample was also collected using 50 mL specimen containers and assessed using the Roche Integra 800 colorimetric assay (Roche Diagnostics Ltd., Rotkreuz, ZG, Switzerland) to determine urinary albumin-creatinine ratio (UACR in mg/mmol). DKD was defined as eGFR <60 mL/min/1.73 m^2^ (*N* = 81) [29], and/or UACR >3.39 mg/mmol (equivalent to 30 mg/g) (*N* = 122) in this study. The CKD-EPI formula has been validated extensively in Asian populations with accuracy of estimating CKD similar to that reported in Caucasian studies [30,31].

### 2.4. Assessment of Covariates

A standardized questionnaire was used to collect information on patients’ demographic and socioeconomic characteristics (e.g., age, gender, income, education), lifestyle factors (e.g., smoking), and medical history (e.g., duration of diabetes, presence of cardiovascular disease (CVD, defined as self-reported history of angina, stroke and myocardial infarction)). A digital BP machine (Dinamap Pro 100 V2, GE Heathcare, Buckinghamshire, UK) was used to perform blood pressure (BP) measurements. Non-fasting venous blood samples (50 mL) were collected by a trained nurse to assess HbA1C, serum total cholesterol, high-density lipoprotein cholesterol (HDL), low-density lipoprotein cholesterol (LDL), and triglycerides. Serum total cholesterol, HDL, LDL and triglycerides were assessed via spectrophotometry conducted using the Beckman Coulter Unicel DxC 800 (Beckman Coulter Inc., Brea, CA, USA), while HbA1C was quantified using immunoassay conducted with the Roche CobasC501 (Roche Diagnostics) [32]. ll samples were analysed at the Singapore General Hospital Hematology Laboratory. Presence of diabetic retinopathy (DR) and diabetic macular edema (DME) in the worst eye was graded from 2-field fundus photographs (Canon CR6-45 NM; Canon Inc., Tokyo, Japan) using the modified Airlie House classification system and confirmed with central macular thickness scans of ≥6 signal strength taken using spectral-domain optical coherence tomography (SD-OCT—Cirrus Version 3.0; Carl Zeiss Meditec, Jena, Germany). Hypertension was defined as having a systolic BP (SBP) of ≥140 mm Hg or a diastolic BP (DBP) of ≥90 mmHg or self-reported history of hypertension or anti-hypertensive medication use.

### 2.5. Statistical Analysis

All statistical analyses were performed using Intercooled Stata version 14.2 for Windows (StataCorp., Lake Station, TX, USA). Patients’ characteristics with and without DKD were compared using the Chi-square statistic for proportions, and a *t* test and/or Mann-Whitney *U* test for means as appropriate to the observed distribution of continuous variables. Normality was checked, and variables were transformed as appropriate. Multivariable binary logistic regression models were used to assess the associations of BMI, WC, WHR and WHtR with the presence of DKD. The exposures were analysed continuously (per SD increase) and categorically (based on the WHO cut-points for BMI and WC; and in quantiles based on the median values for WHR and WHtR). Two models were developed for each exposure, initially including age and gender (Model 1) and additionally for known risk factors of DKD (ethnicity, smoking, presence of CVD, diabetes duration, HbA1C, SBP, BMI, total cholesterol to HDL ratio, presence of DR, use of anti-hypertensive medication, and insulin use) (Model 2) using backward-stepwise variable selection, with threshold significance level for variable removal specified at 0.05. Supplementary subgroup analyses were also conducted for DKD categorized using UACR and eGFR alone to assess the impact of obesity measures on non-albuminuric DKD. Analyses for DKD severity were not undertaken in overall and subgroup analyses; the former because there are no established severity levels for DKD presence defined using both UACR and eGFR values, and the latter due to the small number of patients with more severe disease (*N* = 2 for macroalbuminuria [UACR > 33.9 mg/mmol (equivalent to 300 mg/g)); *N* = 14 for eGFR between 15–29 mL/min/1.73 m^2^; and *N* = 8 for eGFR <15 mL/min/1.73 m^2^).

As previous work [17,21] have demonstrated gender-specific differences in the associations of the different measures of obesity with cardio-metabolic outcomes (e.g., DR and DKD), gender-stratified analyses were conducted. We further considered stratified analyses to isolate the relationships between generalized and abdominal obesity only with DKD. However, the above was not possible due to the small numbers of these individuals (*N* = 35 and *N* = 38 for individuals with generalized and abdominal obesity only, respectively). We instead adjusted for these parameters in the respective analyses (i.e., adjusting for WC in the BMI-DKD relationship and adjusting for BMI in the abdominal obesity-DKD associations) to account for the mutually confounding effects (Table S1). A *p* value of <0.05 was deemed statistically significant.

### 2.6. Meta-Analysis

**Figure 1:**
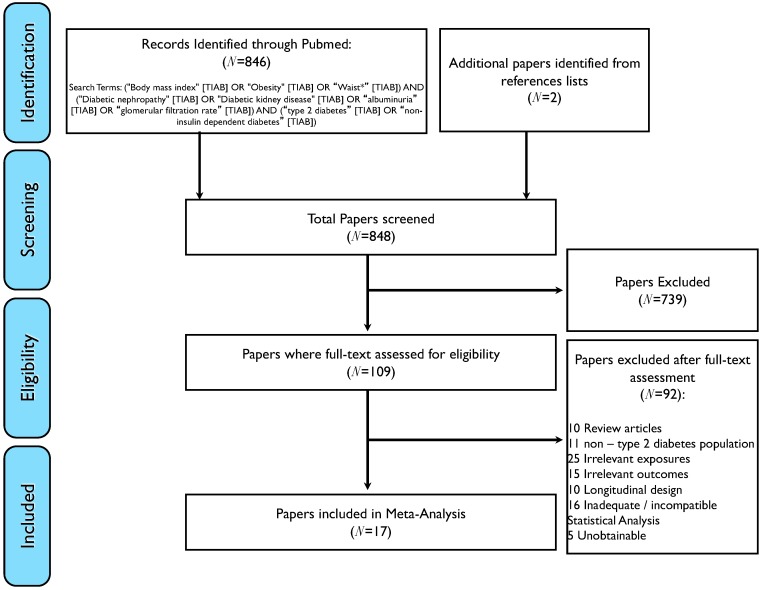
Flowchart showing search terms and article selection for meta-analysis.

The meta-analysis was conducted in accordance with the MOOSE guidelines. To summarize results from the present and previous cross-sectional studies evaluating the relationship between BMI and WHR/WHtR with DKD in persons with T2DM, relevant English-language peer-reviewed clinic- and population-based studies were systematically identified using an electronic literature search of Medline until 31 May 2018, with a combination of keywords (Figure 1) and by scanning relevant reference lists. Previous research assessing the association of generalized obesity and/or abdominal obesity using objectively assessed BMI; and WC or WHR/WHtR, respectively, with DKD (categorized using UACR and/or eGFR) were included. In addition, studies evaluating the categorical BMI- and waist circumference/hip/height ratio-DKD relationships had to specify a cut-off for obesity, instead of simply categorizing and comparing the effects from different quantiles of measurement, due to the need for a common measurement unit when estimating the pooled effect of the exposure on the outcome. This search strategy identified 17 studies (Figure 1).

Random effects meta-analysis was performed to synthesize study effects and heterogeneity was quantified using the I-squared statistic, as substantial variability across studies, due to non-standardized cut-offs for variable categorization being used, was expected. A higher I-squared value meant greater heterogeneity in study effects. All results were expressed in odds ratios (OR) per unit increase for continuous variables, and categorically as obese versus non-obese (for BMI and waist circumference exposures) and per category increase (for waist hip/height ratio exposures). With multiple ORs for waist-hip/height ratio reported in the studies by Wang et al. [33] and Hu et al. [16]. selected effects from each study were averaged prior to incorporating the pooled effects in the meta-analysis. Specifically, in the study by Wang and associates, the per-quartile effects corresponding to definitions of DKD based on albuminuria and eGFR were averaged. In the study by Hu and colleagues, a per-category effect by averaging the effects comparing tertile 2 vs. 1 and tertile 3 vs. 2 was obtained. To obtain standard error estimates of the averaged effects which account for statistical dependency between reported effects [34], model-based estimates of correlation between the reported effects in each study using a logistic regression analysis of outcome frequency counts that were reported was recorded. Sensitivity of the meta-analysis results to varying correlation was checked. Other methods that may incorporate multiple correlated effects directly in the meta-analysis were considered, but the number of waist-hip ratio studies was too small to make valid inferences when employing such methods [35,36].

## 3. Results

The mean age (SD) of the 405 patients included in analyses was 58 (7.5) years and 277 (68.4%) were male. The mean (SD) BMI was 26.5 (4.2) kg/m^2^, 93.3 (10.6) cm for WC, 0.6 (0.1) for WHtR, and 0.9 (0.1) for WHR. Those with DKD comprised 203 (50.1%) of the sample and were likely to be older, male, have a higher total to HDL cholesterol ratio, higher UACR, longer duration of diabetes, higher SBP, greater WHR, and were more likely to be on insulin and have DR (all *p* < 0.05; Table 1).

In models adjusted for age and gender (Table 2, Model 1), no associations were found between BMI analyzed continuously and presence of DKD. However, those categorized as overweight/obese were more likely to have DKD (OR: 1.69, 95% CI: 1.12, 2.55), compared to normal/underweight individuals and this association persisted after multivariable adjustment (Table 2, Model 2). No associations were, however, found between any of the remaining abdominal obesity parameters (WC, WHR and WHtR) with DKD. As being underweight has been found to be associated with DKD in some studies [37,38] we conducted additional Supplementary analyses excluding underweight individuals but found no change in the direction, nor significance of the above reported associations (data not shown). In addition, as iterated previously, we reran the analyses for WHR and WHtR using established abdominal obesity thresholds and did not find any change in the direction, nor significance, of the associations (Table S1). Mutually adjusting for generalized and abdominal obesity exposures showed results similar to that presented in our main tables (Table S1).

In models stratified by gender, we found no association of any of the generalized obesity parameters with DKD (Table 3).

**Figure 2:**
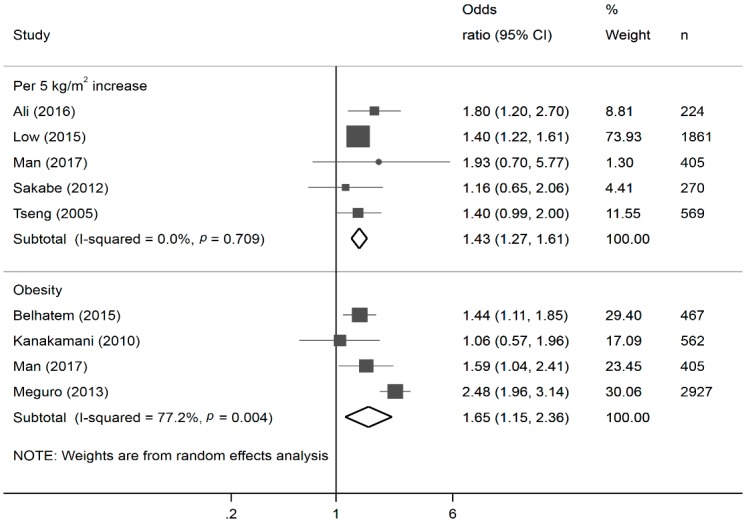
Forest plots * of the association of continuous and categorical body mass index (BMI) with diabetic kidney disease. * The size of the box of each study effect corresponds to the relative weight given to that study in the meta-analysis; the diamond refers to the overall pooled estimates with 95% confidence interval.

The meta-analysis synthesized data from 18 studies (including the present one) for a total of 19,755 participants (Table S2) [11,12,13,14,15,16,17,33,39,40]. For the effects of continuous BMI and obesity (dichotomized BMI) on DKD, we included data from five [11,13,17,40] and four [12,14,39] studies in the meta-analysis, respectively. We found that every 5 kg/m^2^ increase in BMI was on average associated with a 43% increase in the odds of DKD (OR: 1.40, 95% CI: 1.27, 1.61, I-squared: 0%), while obesity was associated with a 65% increase in the odds of renal disease (OR: 1.65, 95% CI: 1.15, 2.34, I-squared: 77.2%; Figure 2).

**Figure 3:**
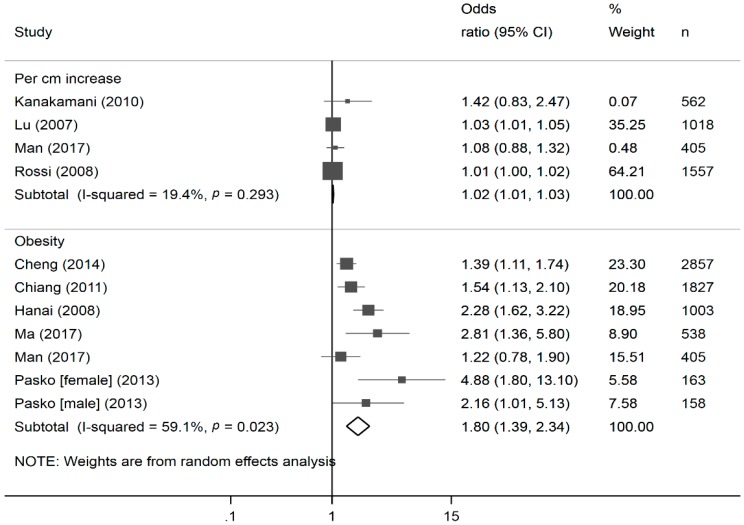
Forest plots * of the association of continuous and categorical waist circumference with diabetic kidney disease. * The size of the box of each study effect corresponds to the relative weight given to that study in the meta-analysis; the diamond refers to the overall pooled estimates with 95% confidence interval.

A total of 4 and 6 studies were included in the meta-analysis of the effects of continuous waist circumference [39,41,42] and abdominal obesity [43,44,45,46,47] (dichotomized waist circumference), respectively. A 1 cm increase in waist circumference was on average associated with a 2% increase in the odds of renal disease (OR: 1.02, 95% CI: 1.01, 1.03, I-squared: 19.4%), while abdominal obesity was associated with an 80% increase in the odds of renal disease (OR: 1.80, 95% CI: 1.39, 2.34, I-squared: 59.1%; Figure 3).

**Figure 4:**
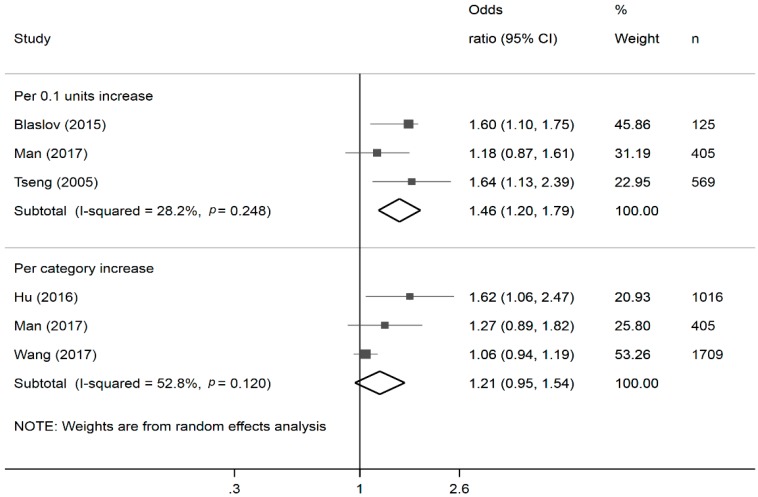
Forest plots * of the association of continuous and categorical waist hip/height ratio with diabetic kidney disease. * The size of the box of each study effect corresponds to the relative weight given to that study in the meta-analysis; the diamond refers to the overall pooled estimates with 95% confidence interval.

Data from six studies (three each) were included in the meta-analysis of the effects of continuous [15,17] and categorized [16,33] WHR/WHtR. While we found a significant association between increased waist-hip ratio and likelihood of DKD continuously (OR per 0.1-unit increase: 1.47, 95% CI 1.25, 1.74, I-squared: 28.2%), this association became attenuated when analyzed categorically (OR per category increase: 1.10, 95% CI 0.99, 1.23, I-squared: 52.8%; Figure 4).

## 4. Discussion

In our clinical study of Asian patients with T2DM, higher BMI was independently associated with greater likelihood of having DKD. WC, WHR and WHtR however, were not independently correlated with DKD presence. While we also found that BMI (i.e., generalised obesity) was associated with greater odds of DKD on our meta-analysis, other parameters of abdominal obesity namely WC, WHR and WHtR, were also associated with a higher likelihood of having DKD. Taken together, our results suggest that both generalized and abdominal obesity may play a role in the pathophysiology of DKD in T2DM, independent of their established roles as major risk factors of hypertension and diabetes, both of which in turn have been demonstrated to be associated with DKD [9]. As such, public health interventions to reduce both forms of obesity in patients with diabetes may also help reduce the likelihood of developing DKD although longitudinal data are needed to support this claim.

Unlike previous research in T2DM patients showing an independent association between abdominal obesity and DKD, as evident in the overall pooled estimates from our meta-analyses, we found no significant relationship between WC, WHR or WHtR and the presence of DKD in our clinical study. Discrepancies between findings could be related to the presence of non-albuminuric DKD, which can make up approximately 50% of individuals with T2DM and DKD [22]. Analyses stratified by classification of DKD (via eGFR or UACR alone) appear to support this theory: the effect sizes for the association of abdominal obesity markers with DKD appear to be larger for DKD categorized using UACR (Table S3) alone versus DKD categorized using eGFR only (Table S4). As such, larger, longitudinal studies are needed to validate our findings.

We demonstrated that being overweight/obese was associated with increased odds of having DKD in both our sample and meta-analysis of cross-sectional studies, despite the high degree of heterogeneity in the meta-analysis (I-squared value of 76.5%). This heterogeneity is likely due to the difference in the classification of obesity and DKD utilized in the various studies; for instance, Belhatem and colleagues defined obesity as BMI of 30 to <40 kg/m^2^, while Low and associates categorized obesity as >25 kg/m^2^. Our cross-sectional results are corroborated by data from large-scale prospective studies [48]. For instance, the Hypertension Detection and Follow-Up Program, a cohort study comprising 5897 patients with hypertension and no kidney disease at baseline, found that the 5-year incidence of kidney disease was 20% higher in obese patients compared to those with normal BMI, even after adjustment for presence of T2DM [48]. Unfortunately, we were unable to assess the relationship between BMI and the more severe stages of DKD as we had too few cases of severe DKD. This is important as a few studies have reported that higher BMI is associated with greater rates of survival in those with ESRD [49,50]; a finding that has been attributed to the phenomenon known as the “obesity paradox” [51], where persons with larger body mass are also likely to be healthier (greater muscle mass) with less comorbid conditions. Hence objective measures of body fat (e.g., using dual-energy X-ray absorptiometry or Magnetic Resonance Imaging) [52] in order to enhance our understanding of the nature of the generalized obesity-DKD relationship are warranted.

As our participants were Asian, we also analysed the BMI-DKD relationship using the Asian cut-points defined by WHO in 2003 (Table S5). We found that although the direction of the association remained similar, the significance became attenuated. This attenuation may indicate the existence of a threshold beyond which a higher BMI contributes significantly to the pathogenesis of DKD, and that this threshold may lie closer to the international than Asian classification of generalized obesity. Further longitudinal studies are warranted to verify our findings.

The mechanisms that underlie the relationship between obesity and DKD, independent of BP and diabetes, are still poorly understood. One hypothesis is that obesity-induced glomerular hyperfiltration, consequent to increased renal tubular sodium reabsorption, results in impairment of renal autoregulation, which then allows for any increase in systemic BP to be transmitted directly to the glomerulus, leading to subsequent renal insult [53]. Excessive lipid deposition into the kidney as a result of obesity can also lead to accumulation of toxic metabolites derived from fatty acid metabolism, e.g., diacylglycerols, resulting in mitochondrial dysfunction, endoplasmic reticulum stress, apoptosis, and eventual renal dysfunction [54].

Strengths of this study include a large clinical sample, a comprehensive and standardized clinical assessment protocol, as well as the use of meta-analysis to synthesize available data on the association between the relevant exposures (BMI, WC, WHR and WHtR) and outcome (DKD). Limitations include the cross-sectional nature of this study limiting causal inferences, as well as the low number of subjects with severe DKD, particularly women, making severity analyses non-viable. In addition, our analyses were conducted in a clinical population, which may affect the generalizability of results. Furthermore, UACR and eGFR were assessed using a single spot measurement, which could have led to non-differential misclassification of albuminuria and CKD status, resulting in over or under-reporting of the true prevalence of albuminuria and CKD in these subjects. Additionally, we were unable to verify if the DKD cases in our study were a consequence of diabetes, or of a non-diabetic nature as kidney biopsies were not performed on participants due to the potential risks associated with the procedure. Lastly, we assessed only the relationships between the anthropometric measures of obesity and DKD in our study sample. Future studies should be conducted using objective body fat measures to verify our findings.

## 5. Conclusions

This study and meta-analysis provide evidence that being overweight or obese, as a result of high BMI and/or anthropometric waist measures, was associated with DKD in Asian persons with T2DM. Longitudinal studies with objective assessments of body fat percentage, distribution, location, as well as nature of the renal dysfunction in participants, are warranted to confirm the role of generalized and abdominal obesity in the pathogenesis of DKD. Our results may also inform future clinical trials to determine if objective, rather than anthropometric, markers of obesity are more clinically relevant risk markers of DKD, as well as public health interventions for patients with diabetes to reduce their likelihood of developing DKD.

## Figures and Tables

**Table 1:** Comparison of participant characteristics stratified by presence of diabetic kidney disease (DKD) *.

	Mean (SD) or Number (%)			
Variable	Overall (*N* = 405)	No DKD (*N* = 202)	DKD * (*N* = 203)	*p* ^
Age (years)	58.0 (7.5)	57.0 (7.4)	58.9 (7.5)	0.012
Gender				
Male	277 (68.4)	130 (64.4)	147 (72.4)	0.081
Female	128 (31.6)	72 (35.6)	56 (27.6)	
Race				
Chinese	303 (74.8)	149 (73.8)	154 (75.9)	0.193
Malay	35 (8.6)	14 (6.9)	21 (10.3)	
Indian	67 (16.5)	39 (19.3)	28 (13.8)	
Total to HDL cholesterol ratio	4.2 (1.3)	4.0 (1.1)	4.4 (1.4)	0.004
ACR (mg/mmoL)	22.2 (62.8)	1.3 (0.8)	43.0 (83.8)	<0.001
HbA1C (%)	7.8 (1.6)	7.5 (1.4)	8.1 (1.6)	<0.001
Systolic blood pressure (mmHg)	136.3 (17.8)	132.2 (16.4)	140.5 (18.2)	<0.001
Diastolic blood pressure (mmHg)	77.3 (9.8)	76.4 (9.1)	78.2 (10.5)	0.058
Diabetes duration (years)	13.3 (9.3)	11.1 (8.5)	15.6 (9.5)	<0.001
Insulin use				
No insulin use	347 (85.7)	183 (90.6)	164 (80.8)	0.005
Insulin use	58 (14.3)	19 (9.4)	39 (19.2)	
Presence of DR				
No	178 (44.0)	106 (52.5)	72 (35.5)	0.001
Yes	227 (56.0)	96 (47.5)	131 (64.5)	
Anti-hypertensive medication use				
No	259 (64.0)	137 (67.8)	122 (60.1)	0.106
Yes	146 (36.0)	65 (32.2)	81 (39.9)	
Generalized Obesity categories				
Normal or underweight (BMI < 25 kg/m^2^)	168 (41.5)	94 (46.5)	74 (36.5)	0.104
Overweight (BMI 25–29.9 kg/m^2^)	160 (39.5)	71 (35.1)	89 (43.8)	
Obese (BMI ≥ 30 kg/m^2^)	77 (19.0)	37 (18.3)	40 (19.7)	
BMI (kg/m^2^)	26.5 (4.2)	26.3 (4.3)	26.7 (4.1)	0.331
Abdominal Obesity categories				
Normal or underweight	127 (31.4)	66 (32.7)	61 (30.0)	0.569
Overweight or obese (waist circumference >90 cm for males; >80 cm for females	278 (68.6)	136 (67.3)	142 (70.0)	
Waist circumference (cm)	93.3 (10.6)	92.7 (10.7)	94.0 (10.4)	0.206
Waist-hip ratio quantiles				
Lower quantile (0.72–0.94)	203 (50.1)	112 (55.4)	91 (44.8)	0.033
Upper quantile (0.95–1.13)	202 (49.9)	90 (44.6)	112 (55.2)	
Waist-hip ratio	0.9 (0.1)	0.9 (0.1)	1.0 (0.1)	0.017
Waist-height ratio quantiles				
Lower quantile (0.41–0.56)	203 (50.1)	109 (54.0)	94 (46.3)	0.123
Upper quantile (0.57–0.80)	202 (49.9)	93 (46.0)	109 (53.7)	
Waist-height ratio	0.6 (0.1)	0.6 (0.1)	0.6 (0.1)	0.168

^ *t*-test or chi-squared test. * Based on eGFR (<60 mL/min/1.73 m^2^) and urinary albumin creatinine ratio (>3.3 mg/mmol). ACR: albumin-creatinine ratio, HbA1C: haemoglobin A1C, HDL: high density lipoprotein, DR: diabetic retinopathy, BMI: body mass index

**Table 2:** Multivariable adjusted association of body mass index, waist circumference, waist-hip-ratio and waist-height-ratio with diabetic kidney disease * (*N* = 405).

		Odds Ratio (95% CI)			
		Model 1	*p*	Model 2	*p*
Body mass index	Overweight or obese	1.69 (1.12 to 2.55)	0.012	1.59 (1.04 to 2.41)	0.030
	Per SD increase	1.20 (0.97 to 1.47)	0.091	1.14 (0.93 to 1.42)	0.213
Waist circumference	Overweight or obese	1.62 (0.94 to 2.78)	0.084	1.25 (0.70 to 2.24)	0.457
	Per SD increase	1.13 (0.93 to 1.38)	0.228	1.08 (0.88 to 1.32)	0.484
Waist-hip-ratio	Upper quantile (0.95–1.13)	1.39 (0.92 to 2.10)	0.114	1.27 (0.83 to 1.93)	0.271
	Per SD increase	1.23 (0.97 to 1.55)	0.086	1.14 (0.90 to 1.45)	0.281
Waist-height-ratio	Upper quantile (0.57–0.80)	1.41 (0.95 to 2.10)	0.091	1.28 (0.85 to 1.92)	0.239
	Per SD increase	1.18 (0.97 to 1.45)	0.099	1.11 (0.91 to 1.37)	0.304

Model 1: Age and gender. Model 2: Model 1 + ethnicity, smoking, presence of cardiovascular disease, diabetes duration, HbA1c, systolic blood pressure, total cholesterol to high density cholesterol ratio, presence of retinopathy, use of anti-hypertensive medication, and insulin use using stepwise regression. * Based on eGFR (<60 mL/min/1.73 m^2^) and/or urinary albumin creatinine ratio (>3.39 mg/mmol). SD: standard deviation.

**Table 3:** Multivariable * adjusted and gender-stratified associations of body mass index, waist circumference, waist-hip-ratio and waist-height-ratio with diabetic kidney disease (*N* = 405).

		Male		Female	
		Odds Ratio (95% CI)	*p*	Odds Ratio (95% CI)	*p*
Body mass index	Overweight or obese	1.45 (0.88 to 2.39)	0.149	1.88 (0.86 to 4.13)	0.115
	Per SD increase	1.08 (0.82 to 1.42)	0.581	1.23 (0.87 to 1.73)	0.240
Waist circumference	Overweight or obese	1.45 (0.88 to 2.37)	0.141	1.69 (0.56 to 5.11)	0.356
	Per SD increase	0.71 (0.42 to 1.18)	0.188	1.35 (0.91 to 1.98)	0.133
Waist-hip-ratio	Upper quantile (0.95–1.13)	1.12 (0.68 to 1.85)	0.662	1.71 (0.78 to 3.73)	0.181
	Per SD increase	0.99 (0.70 to 1.39)	0.932	1.41 (0.93 to 2.14)	0.106
Waist-height-ratio	Upper quantile (0.57–0.80)	1.13 (0.69 to 1.85)	0.632	1.68 (0.81 to 3.50)	0.164
	Per SD increase	1.08 (0.62 to 1.85)	0.794	1.18 (0.83 to 1.69)	0.360

* Adjusted for age, gender, ethnicity, smoking, presence of cardiovascular disease, diabetes duration, HbA1c, systolic blood pressure, total cholesterol to high density cholesterol ratio, presence of retinopathy, use of anti-hypertensive medication, and insulin use using stepwise regression. * Based on eGFR (<60 mL/min/1.73 m^2^) and/or urinary albumin creatinine ratio (>3.39 mg/mmoL). SD: standard deviation

## Supplementary Materials

The following are available online at http://www.mdpi.com/2072-6643/10/11/1685/s1, Table S1: Multivariable * adjusted association of body mass index, waist circumference, waist-hip-ratio and waist-height-ratio with diabetic kidney disease† (*N* = 405), Table S2: Summary of included studies in meta-analysis, Table S3: Multivariable adjusted association of body mass index, waist circumference, waist-hip-ratio and waist-height-ratio with diabetic kidney disease * (*N* = 405), Table S4: Multivariable adjusted association of body mass index, waist circumference, waist-hip-ratio and waist-height-ratio with diabetic kidney disease * (*N* = 405), Table S5: Multivariable adjusted association of body mass index with diabetic kidney disease using Asian BMI obesity cut-points * (*N* = 405).
