# Supplementary material for: The Relationship between Generalized and Abdominal Obesity with Diabetic Kidney Disease in Type 2 Diabetes: A Multiethnic Asian Study and Meta-Analysis
Source: Nutrients. 2018 Nov 5;10(11):1685. doi: 10.3390/nu10111685 (PMC6266073; doi:10.3390/nu10111685)
Supplement: Supplementary file 1 [file nutrients-10-01685-s001.pdf]

Table S1. Multivariable* adjusted association of body mass index, waist circumference, waist-hip-ratio and waist-height-ratio with diabetic kidney disease† (N = 405).

		Both genders		Male		Female	
		Odds ratio (95% CI)	*P*	Odds ratio (95% CI)	*P*	Odds ratio (95% CI)	*P*
Body mass index	Overweight or obese	1.96 (1.10 to 3.47)	0.021	2.28 (1.10 to 4.74)	0.027	1.54 (0.58 to 4.03)	0.384
	Per SD	1.28 (0.86 to 1.90)	0.221	1.51 (0.85 to 2.67)	0.157	0.98 (0.55 to 1.74)	0.946
Waist circumference	Overweight or obese (> 94 (M), > 80 (F))	1.03 (0.58 to 1.83)	0.928	0.93 (0.45 to 1.91)	0.838	1.79 (0.52 to 6.16)	0.354
	Per SD	0.88 (0.60 to 1.29)	0.510	0.71 (0.42 to 1.18)	0.188	1.37 (0.72 to 2.61)	0.340
	Per SD	0.88 (0.60 to 1.29)	0.510	0.71 (0.42 to 1.18)	0.188	1.37 (0.72 to 2.61)	0.340
Waist-hip-ratio	Overweight or obese (≥ 0.90 (M), ≥ 0.85 (F))	0.88 (0.50 to 1.53)	0.646	0.81 (0.38 to 1.73)	0.592	1.10 (0.46 to 2.63)	0.824
	Per SD	1.10 (0.85 to 1.42)	0.485	0.99 (0.70 to 1.39)	0.932	1.39 (0.91 to 2.11)	0.128
Waist-height-ratio	Overweight or obese (> 0.5)	0.74 (0.38 to 1.45)	0.377	0.69 (0.32 to 1.52)	0.361	1.01 (0.24 to 4.21)	0.984
	Per SD	0.99 (0.67 to 1.46)	0.951	1.08 (0.62 to 1.85)	0.794	0.99 (0.54 to 1.80)	0.966

*Adjusted for age, gender, total to high density lipoprotein cholesterol ratio and insulin use. † Based on eGFR (<60) and microalbuminuria (>3.3). BMI models were adjusted for waist circumference and waist models were adjusted for BMI

Table S2. Summary of included studies in meta-analysis.

Author	Year	Title	Country	Sample Size	Exposure	Exposure Definition	Outcome Definition
Tseng et al.	2005	Waist-to-height ratio is independently and better associated with urinary albumin excretion rate than waist circumference or waist-to-hip ratio in chinese adult with type 2 diabetic women but not men.	Taipei, Taiwan	569	BMI (Per kg/m²increase), Waist-height ratio (per 0.1-unit increase)	BMI = Weight (kg) divided by height squared,<br>Waist-height ratio = Waist Circumference divided by body height	UACR > 30ug/mg
Lu et al.	2007	High prevalence of albuminuria in population-based patients diagnosed with type 2 diabetes in the Shanghai downtown	Shanghai, China	1039	Waist circumference (in cm)	Waist circumference (per cm increase)	UACR 30–299mg/g
Rossi et al.	2008	Identifying patients with type 2 diabetes at high risk of microalbuminuria: results of the DEMAND (Developing Education on Microalbuminuria for	Italy	1841	Waist circumference (in cm)	Waist circumference (per 5 cm increase)	UACR 30–299mg/g

		Awareness of reNal and cardiovascular risk in Diabetes) Study					
Hanai et al.	2008	Renal manifestations of metabolic syndrome in type 2 diabetes	Japan	1003	Central obesity yes Vs no	Waist circumference ≥85 cm for men and ≥ 90 cm for women	UACR 30–299mg/g
Kanakamani et al.	2010	Prevalence of microalbuminuria among patients with type 2 diabetes mellitus--a hospital-based study from north India	North India	562	Obese Vs Not Obese & waist circumference (per cm increase)	BMI ≥ $25kg/m^2$ & waist circumference	Change in color of Micral 2 strip that corresponds to UACR >20mg/L
Chiang et al.	2011	Justifying the high prevalence of microalbuminuria for type 2 diabetic patients in Taiwan with conditional probability approach--a DEMAND II study	Taiwan	1924	Abnormal Vs normal waist circumference	Waist circumference >90 cm (men) or >80 cm (women)	UACR ≥ 30mg/g
Sakabe et al.	2012	Low daily salt intake is correlated with albuminuria in patients with type 2 diabetes	Kyoto, Japan	270	BMI (Per kg/m^2 increase)	Weight (kg) divided by height squared	UACR (Urine Albumin-Creatinine Ratio) > 30mg/g
Meguro et al.	2013	Past Obesity as well as Present Body Weight Status Is a Risk Factor for Diabetic Nephropathy	Tokyo, Japan	2927	Obese Vs Not Obese	BMI ≥ $25kg/m^2$	UAER (Urinary Albumin Excretion Rate) >20ug/min
Pasko et al.	2013	Prevalence of microalbuminuria and risk factor analysis in type 2 diabetes patients in Albania: the need for accurate and early diagnosis of diabetic nephropathy	Albania	321	Abnormal Vs normal waist circumference	Waist circumference ≥ 102 cm for males or ≥ 88 cm for females	UACR ≥ 30mg/g
Cheng et al.	2014	Cardiometabolic risk profiles associated with chronic complications in overweight and obese type 2 diabetes patients in South China	Guangdong, China	2954	Abdominal obesity yes Vs no	Waist circumference >90 cm (men) or >80 cm (women)	UAER: ≥ 20mg/min
Blaslov et al.	2015	Waist-to-height ratio is independently associated with chronic kidney disease in overweight type 2 diabetic patients	Croatia	125	Waist-height ratio (per 0.1-unit increase)	Waist Circumference divided by body height	eGFR (estimated glomerular filtration rate) < 60mL/min per $1.73m^2$ AND/OR UACR >30mg/24hr

Low et al.	2015	Prevalence of Chronic Kidney Disease in Adults with Type 2 Diabetes Mellitus	Singapore	1861	BMI (Per kg/m^2 increase)	Weight (kg) divided by height squared	eGFR < 60ml/min per 1.73 m^2 AND/OR UACR > 30mg/g
Belhatem et al.	2015	Impact of morbid obesity on the kidney function of patients with type 2 diabetes	Paris, France	467	Obese Vs Not Obese	BMI ≥ 25kg/m^2	UACR > 30mg/g
Ali et al.	2016	Prevalence and determinants of microalbuminurea among type 2 diabetes mellitus patients, Baghdad, Iraq, 2013	Iraq	224	BMI (Per kg/m^2 increase)	Weight (kg) divided by height squared	UACR 30–300mg/g
Hu et al.	2016	Abdominal Obesity Is More Closely Associated With Diabetic Kidney Disease Than General Obesity	Chongqing, China	1016	Waist-height ratio (per quantile increase)	Waist Circumference divided by body height	eGFR < 60mL/min per 1.73m^2
Wang et al.	2017	Abdominal adiposity contributes to adverse glycemic control and albuminuria in Chinese type 2 diabetic patients: A cross-sectional study	Shanghai, China	1709	Waist-hip ratio (per quantile increase)	Wasit Circumference divided by hip circumference	UACR ≥ 10.18mg/g
Ma et al.	2017	The Relationship between Hypertriglyceridemic Waist Phenotype and Early Diabetic Nephropathy in Type 2 Diabetes	Qinhuangdao, China	538	Hypertriglycermic waist phenotype yes Vs no	Serum TG concentrations ≥1.7 mmol/L and waist circumference ≥ 90 cm (males) and ≥ 85 cm (females)	Urinary microalbumin (UMA) 20–200 μg/min
Man et al.	2018	Relationship between Generalized and Abdominal Obesity with Diabetic Kidney Disease in Type 2 Diabetes: A Multiethnic Asian Study and Meta-Analysis	Singapore	405	BMI: Per kg/m^2 increase & overweight/obese versus under/normal weight, Waist circumference: per cm increase & abdominal obesity versus non-abdominal obesity, Waist-hip/height ratio: per 0.1 unit	BMI = Weight (kg) divided by height squared,<br><br>Waist-hip ratio = Waist Circumference divided by hip circumference<br><br>Waist-height ratio = Waist circumference divided by height	UACR > 3.3 mg/mmoL AND/OR<br>eGFR < 60 ml/min per 1.73 m^2

increase & by median

Table S3. Multivariable adjusted association of body mass index, waist circumference, waist-hip-ratio and waist-height-ratio with diabetic kidney disease* ($N = 405$).

		Odds ratio (95% CI)			
		**Model 1**	***P***	**Model 2**	***P***
Body mass index	Overweight or obese	1.76 (1.17 to 2.66)	0.007	1.65 (1.09 to 2.51)	0.019
	Per SD increase	1.21 (0.98 to 1.49)	0.071	1.16 (0.94 to 1.44)	0.159
Waist circumference	Overweight or obese	1.33 (0.85 to 2.06)	0.210	1.22 (0.78 to 1.91)	0.389
	Per SD increase	1.21 (0.99 to 1.48)	0.066	1.16 (0.95 to 1.43)	0.154
Waist-hip-ratio	Upper quantile (0.95–1.13)	1.67 (1.10 to 2.52)	0.016	1.54 (1.01 to 2.35)	0.044
	Per SD increase	1.30 (1.03 to 1.65)	0.029	1.23 (0.97 to 1.57)	0.093
Waist-height-ratio	Upper quantile (0.57–0.80)	1.56 (1.05 to 2.32)	0.029	1.45 (0.96 to 2.17)	0.076
	Per SD increase	1.23 (1.00 to 1.50)	0.048	1.17 (0.95 to 1.43)	0.142

Model 1: Age and gender. Model 2: Model 1 + ethnicity, smoking, presence of cardiovascular disease, diabetes duration, HbA1 c, systolic blood pressure, total cholesterol to high density cholesterol ratio, presence of DR, use of anti-hypertensive medication, and insulin use using stepwise regression. * Based on urinary albumin creatine ratio (> 3.39 mg/mmol) only.

Table S4. Multivariable adjusted association of body mass index, waist circumference, waist-hip-ratio and waist-height-ratio with diabetic kidney disease* ($N = 405$).

		Odds ratio (95% CI)			
		**Model 1**	***P***	**Model 2**	***P***
Body mass index	Overweight or obese	2.07 (1.21 to 3.56)	0.008	1.86 (1.05 to 3.29)	0.033
	Per SD increase	1.25 (0.95 to 1.64)	0.105	1.14 (0.85 to 1.52)	0.386
Waist circumference	Overweight or obese	1.91 (1.07 to 3.43)	0.029	1.66 (0.90 to 3.08)	0.105
	Per SD increase	1.17 (0.90 to 1.51)	0.243	1.05 (0.79 to 1.39)	0.738
Waist-hip-ratio	Upper quantile (0.95 – 1.13)	1.03 (0.61 to 1.74)	0.900	0.84 (0.48 to 1.46)	0.543
	Per SD increase	1.15 (0.85 to 1.56)	0.368	0.97 (0.70 to 1.35)	0.855

Waist-height-ratio	Upper quantile (0.57 – 0.80)	1.75 (1.05 to 2.92)	0.033	1.44 (0.84 to 2.48)	0.187
	Per SD increase	1.21 (0.94 to 1.58)	0.145	1.06 (0.80 to 1.40)	0.671

Model 1: Age and gender. Model 2: Model 1 + ethnicity, smoking, presence of cardiovascular disease, diabetes duration, HbA1c, systolic blood pressure, total cholesterol to high density cholesterol ratio, presence of DR, use of anti-hypertensive medication, and insulin use using stepwise regression. * Based on eGFR (<60 mL/min/1.73m^2) only.

Table S5. Multivariable adjusted association of body mass index with. diabetic kidney disease using Asian BMI obesity cut-points* (*N* = 405).

		**Both genders**		**Male**		**Female**	
		Odds ratio (95% CI)	*P*	Odds ratio (95% CI)	*P*	Odds ratio (95% CI)	*P*
Body mass index	Overweight or obese (Asian)	1.30 (0.79 to 2.13)	0.295	1.31 (0.74 to 2.32)	0.350	1.28 (0.47 to 3.47)	0.632
	Per SD	1.14 (0.93 to 1.42)	0.213	1.08 (0.82 to 1.42)	0.581	1.23 (0.87 to 1.73)	0.240

*Adjusted for age, gender, total to high density lipoprotein cholesterol ratio and insulin use. † Based on eGFR (< 60) and microalbuminuria (> 3.3).
